# The Value of Body Plethysmography (sGaw) in the Assessment of Airway Hyperreactivity in Cough Variant Asthma

**DOI:** 10.3390/jcm14010074

**Published:** 2024-12-27

**Authors:** Natasa Karamarkovic Lazarusic, Sanja Popovic-Grle, Ena Tolic, Anamarija Stajduhar, Renata Bozinovic, Gordana Pavlisa

**Affiliations:** 1 Outpatient Centre for Respiratory Diseases, 10000 Zagreb, Croatia; natasa.karamarkovic@yahoo.com; 2 Department for Respiratory Diseases Jordanovac, University Hospital Centre Zagreb, 10000 Zagreb, Croatia; sanja.popovic.grle@kbc-zagreb.hr (S.P.-G.); ena.tolic1@gmail.com (E.T.); stajduhar.anamarija@gmail.com (A.S.); 3 School of Medicine, University of Zagreb, 10000 Zagreb, Croatia; 4 University Hospital Centre Osijek, 31000 Osijek, Croatia; rena7ster@gmail.com

**Keywords:** cough variant asthma, methacholine challenge test, plethysmography, specific conductance, diagnostic accuracy, sensitivity, specificity

## Abstract

**Background/Objectives**: Cough variant asthma (CVA) is characterized by nonspecific symptoms and normal spirometric values, which makes diagnosis challenging. To diagnose CVA it is necessary to document airway hyperreactivity (AHR). The aim of our study was to evaluate the diagnostic value of body plethysmography in the assessment of AHR using the methacholine challenge test (MCT). **Methods**: In CVA-suspected patients, a bronchodilation test (BDT), an MCT with spirometry, and body plethysmography were performed. The MCT was considered positive if there was a 20% decrease in forced expiratory volume in 1 s from the baseline value (PC_20_FEV_1_) or a 40% reduction in specific conductance (PC_40_sGaw) after inhaling methacholine of concentration < 8 mg/mL. Sensitivity and specificity were generated for different cut off points of sGaw (PC_40_sGaw, PC_45_sGaw, PC_50_sGaw). Anti-asthma treatment was started for those with proven AHR. The diagnosis of asthma was made after one year of follow-up based on the response to treatment. **Results**: AHR was diagnosed in 83.5% (91/109) of patients by either a BDT, PC_20_FEV_1_, or PC_40_sGaw. After one year of follow-up, asthma was confirmed in 76 patients. The sensitivities of the BDT, PC_20_FEV_1_, and PC_40_sGaw were 25%, 64%, and 97%, respectively. The specificities of the BDT, PC_20_FEV_1_ and PC_40_sGaw were 94%, 88%, and 67%, respectively. The sensitivities for a PC_45_sGaw and PC_50_sGaw were 88% and 63%, and the specificities were 82% and 91%, respectively. **Conclusions**: Body plethysmography is a valuable tool in the assessment of AHR in CVA, with the best sensitivity-to-specificity ratio found at a PC_45_sGaw.

## 1. Introduction

Chronic cough is a common medical problem that affects about 10% of the general adult population [1]. It could be a symptom of numerous pulmonary and extrapulmonary diseases. It is one of the prominent symptoms of asthma, which is one of the leading global burdens. It is estimated that asthma affects about 300 million people worldwide and causes about 1000 deaths a day [2]. Cough variant asthma (CVA) has been recognized as a specific form of asthma characterized by cough as the sole presenting symptom and normal baseline lung function [3,4]. Prospective studies in patients with chronic cough have shown that, on average, 25% of adults with chronic cough have CVA [5]. Due to nonspecific symptoms and normal findings of the basic examination, it can often be misdiagnosed [6]. According to the Global Initiative for Asthma, the diagnosis of asthma should be based on a combination of typical respiratory symptoms and documented airway hyperresponsiveness (AHR) [2]. A widely used test for confirming AHR is a positive bronchodilator test (BDT). The test is highly specific, but its sensitivity for CVA is low since a significant improvement in the forced expiratory volume in 1 s (FEV_1_) is difficult to achieve among individuals with normal baseline lung function [7]. As the next diagnostic step, direct bronchial provocation tests (BPTs), such as the methacholine provocation test, are most often used in clinical practice. For patients who cannot tolerate methacholine or have contraindications, an alternative method is the cold dry air provocation test. However, it has not been widely used due to lack of standardization and variable sensitivity and specificity compared to methacholine [8,9]. Spirometry is the most common method for detecting airway narrowing following methacholine inhalation. Another option for evaluating the response to methacholine is to perform a BPT by body plethysmography. Body plethysmography requires minimal subject cooperation and is performed under conditions of tidal breathing, without deep inhalation and thoracic gas compression. Besides providing information on the change in specific conductance (sGaw), body plethysmography offers additional data, such as changes in lung volumes. Comparative studies between spirometry and body plethysmography in the assessment of BPTs suggest the better sensitivity of body plethysmography [10,11]. Higher sensitivity of plethysmography in comparison with spirometry was first reported by Fish JE et al. in 1976, showing that the response to methacholine of subjects with hay fever was similar to that of healthy subjects when measuring FEV_1_ but similar to that of subjects with asthma when measuring sGaw [12]. Some studies suggest a lower degree of AHR in CVA patients than in a group of patients with classic asthma [13,14]. Therefore, in this group of patients, AHR assessment by body plethysmography could have additional value. Current guidelines do not provide strict recommendations on the change in specific conductance (sGaw) needed to determine AHR [15]. A reduction in sGaw from 35 to 45% (sGaw_35_-sGaw_45_) was used in studies to indicate clinical significance [16,17,18,19].

The aims of our study were as follows

i. Compare spirometry and body plethysmography in order to evaluate their clinical value during BPTs in CVA;
ii. Examine the sensitivity and specificity of a PC_20_FEV_1_ and different reductions in sGaw (PC_40_sGaw, PC_45_sGaw, and PC_50_sGaw) in defining AHR.

## 2. Materials and Methods

### 2.1. Study Design

**Figure 1:**
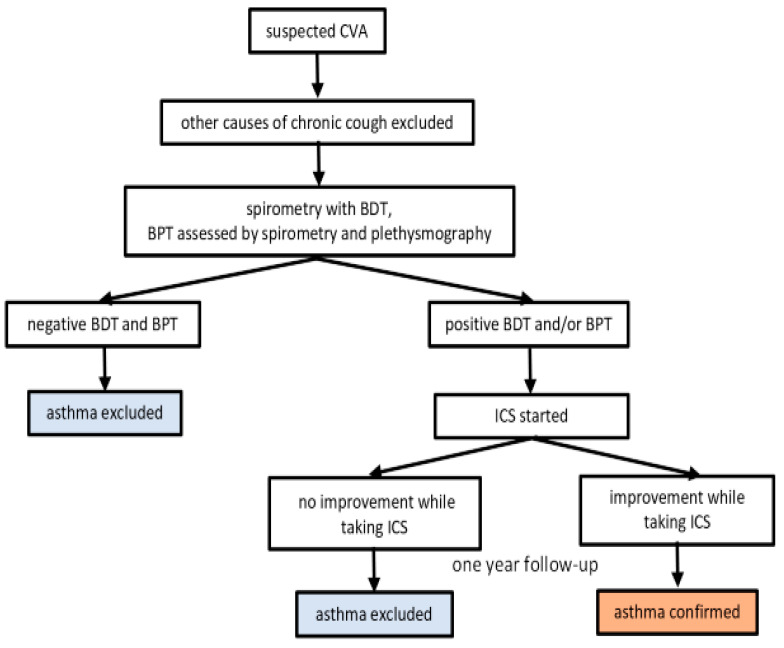
Diagnostic flowchart.

This study included 109 patients referred by primary care physicians for evaluation of chronic cough to the Outpatient Centre for Respiratory Diseases, Zagreb, Croatia. The patients were examined by pulmonologists and suspected of having CVA. The only symptom the patients reported was a cough that lasted at least 8 weeks. The diagnostic procedure consisted of the following: FeNO measurement, spirometry with a BDT, a BPT assessed by spirometry and plethysmography, and a skin prick test for inhalation allergens. For all patients, chest X-rays were performed to exclude other causes of chronic cough. All patients were referred to an otorhinolaryngologist to rule out other causes of cough such as gastroesophageal reflux or postnasal drip. Patients with well-known contraindications for BDTs and BPTs, including unstable coronary artery disease, cardiac arrhythmia, uncontrolled arterial hypertension, untreated hypothyroidism, or pregnancy, were excluded. In patients with signs of respiratory infection, testing was postponed for 6 weeks. Smokers were requested to refrain from smoking for 24 h before testing. If the patient was taking angiotensin-converting enzyme (ACE) inhibitors, testing was continued if cough persisted one month after ACE inhibitor discontinuation. None of the patients had previously been diagnosed with obstructive airway disease, nor were they using anti-asthma medications. Only patients whose baseline spirometric values were within normal limits were included in the study. Patients who met the inclusion and exclusion criteria were consecutively included in the study. In patients with a positive BDT or BPT, treatment with low doses of inhaled corticosteroids (ICS) was started. They were followed-up for one year. Patients who reported cough improvement while taking ICS and who were regularly taking the medicine until the one-year follow-up visit were considered to have asthma (Figure 1). The study was approved by the Ethics Committee of the University Hospital Centre Zagreb, No.: 02/013 AG.

### 2.2. Pulmonary Function Tests

Pulmonary function tests were performed by experienced respiratory technicians.

Spirometry was conducted using the Jaeger MasterScreen device (Jaeger GmbH, Wurzburg, Germany) in line with the 2019 recommendations of the American Thoracic Society (ATS)/European Respiratory Society (ERS) [20]. The forced vital capacity (FVC) and forced expiratory volume in 1 s (FEV_1_) were expressed in liters (L) and as percentages of predicted values (%). Airway obstruction was diagnosed when FEV_1_/FVC was <0.70 and/or FEV_1_ was <80% of the predictive value [21]. Spirometry was performed before and 20 min after the inhaled bronchodilator (salbutamol 400 µg from a pressurized inhaler). An increase of 12% and 200 mL in either FEV_1_ or FVC provided evidence of bronchodilator responsiveness (reversibility) [20].

Body plethysmography was conducted using the Jaeger MasterScreen Body Device (Jaeger GmbH, Wurzburg, Germany) in accordance with standard protocols [22]. Total lung capacity (TLC), residual volume (RV), and specific airway conductance (sGaw) were recorded.

BPTs were performed in accordance with ATS/ERS standards [23]. Subjects inhaled aerosolized normal saline, followed by aerosolized saline containing methacholine, in doubling concentrations ranging from 0.03 to 8.0 mg/mL. The two-minute tidal breathing inhalation method was used. The measurements of whole-body plethysmography and spirometry were taken two minutes after the inhalation of each methacholine concentration, using a body plethysmograph. The test was considered positive if there was a 20% decrease in FEV_1_ from the baseline value (PC_20_FEV_1_) after inhaling methacholine at a concentration < 8 mg/mL. For sGaw, we defined a methacholine challenge test (MCT) as positive based on a provocative methacholine concentration (<8 mg/mL) causing a 40% reduction in sGaw. Since spirometry is a standardized method, the BPT was stopped when there was a drop in FEV_1_ of 20%.

### 2.3. FeNO Measurement

The FeNO level was measured by a Niox Mino device (Aerocrine AB, Solna, Sweden) at a constant flow rate of 50 mL/s for 10 s, in accordance with ATS/ERS recommendations [24]. FeNO was measured three times, with differences in measured values ≤ 10%. The mean values of the three measurements were used as data for statistical analysis. FeNO was measured before pulmonary function and bronchoprovocative challenge testing.

### 2.4. Skin Prick Test

Skin prick testing was conducted per standard clinic protocol. All patients were tested with an aeroallergen screening panel (Diater Laboratorios, Barcelona, Spain) that included the following allergen extracts: Dermatophagoides pteronyssinus, birch, hazel, timothy grass, ragweed, and cat and dog dander. Histamine hydrochloride (1 mg/mL) was used as a positive control and 50% glycerol-saline solution was used as a negative control. All tests were performed on the patients’ forearms by well-trained nurses. The results were documented 20 min later by an experienced investigator. If it was positive for at least one tested allergen (indurate ≥ 3 mm in diameter), the test was considered positive.

### 2.5. Statistical Analysis

Statistical analysis was performed with Statistica software, version 12 (Dell Inc., Tulsa, OK, USA).

The Kolmogorov–Smirnov test was used to assess whether the variables were normally distributed. Categorical data were presented as frequencies and percentages. Normally distributed continuous data were presented as means with their standard deviation (SD), while abnormally distributed data were presented as the median with their quartile deviation (QD).

Differences between the groups for normally distributed continuous data were assessed by a t-test for independent samples, and for abnormally distributed data the Mann–Whitney U test was used. The χ^2^ test was used for the assessment of differences in categorical variables; *p* values < 0.05 were considered significant. Pre- and post-test lung function parameter values in asthmatics and non-asthmatics have been compared with two-way repeated-measures ANOVA and p-values for those interactions have been reported. The diagnostic validity of the test was assessed by sensitivity, specificity, and positive and negative predictive values, and the odds ratios for a positive and a negative test were calculated.

## 3. Results

### 3.1. Study Population

A total of 109 patients suspected of cough variant asthma were included in the study. The median age of the group was 36.0 (26–44) years, with 36 men (33%) and 73 women (67%). There were 16 (14.7%) current smokers and 93 (85.3%) non-smokers and ex-smokers. In 71 (65.1%) patients, the skin prick test was positive for at least one allergen tested, while it was negative in 38 (34.9%) patients. The mean FeNO value for the whole study population was 48.08 ± 40.04 ppb.

### 3.2. Differences Between Asthma and Non-Asthma Group

The group of patients with asthma was older (median 38.0 (28.0–46.5) years) than the non-asthma group (median 28.0 (20.5–46.5) years). The groups did not differ in sex (*p* = 0.478), BMI (*p* = 0.278), smoking habit (*p* = 0.839), or hypersensitivity to the tested allergens (*p* = 0.660).

Although the baseline values of the pulmonary function tests were normal in both groups, FEV_1_, FEV_1_ expressed as a percentage of predicted value (FEV_1_%), FEV_1_/FVC, MEF_50_, MEF_50_ expressed as a percentage of predicted value (MEF50%), sGaw, and sGaw expressed as a percentage of predicted value (sGaw%) were significantly lower in the asthma group. RV and RV expressed as a percentage of predicted value (RV%) were significantly higher in the asthma group. Table 1 shows the baseline data for the asthma and non-asthma groups.

AHR was diagnosed in 83.5% (91/109) of patients using either a BDT, PC_20_FEV_1_, or PC_40_sGaw. After one year of follow-up, asthma was confirmed in 83.5% (76/91) and not confirmed in 16.5% (15/91) of patients with demonstrated AHR. There were 11 smokers in the AHR group, and 5 smokers in the non-AHR group (*p* = 0.18).

FeNO was significantly higher in the asthma group. In the asthma group, the median FeNO value was 41.0 (24.0–77.75) ppb, while in the non-asthma group, the median FeNO was 20.0 (14.5–41.5) ppb (*p* < 0.001).

Patients with and without asthma responded differently to challenges during BDTs and BPTs (PC_20_ and PC40). The asthma group had a significantly higher FEV_1_ (L and %) and FVC (%) in BDTs and a significantly lower FEV_1_ (L) and sGaw (L/kPa/s) in BDTs (Table 2).

### 3.3. Diagnostic Accuracy of Assessing BHR by Using Different Lung Function Tests

In patients with positive BPTs assessed by spirometry and plethysmography, the provocative concentration of methacholine causing a PC_40_sGaw was significantly lower than the methacholine concentration causing a PC_20_FEV_1_ (*p* < 0.001). The median provocative concentration of methacholine that caused a PC_40_sGaw was 0.5 (0.13–1) mg/mL, and the median PC_20_FEV_1_ was 1 (0.5–4) mg/mL

In the studied group, the BDT was positive in 21 (19.27%), PC_20_FEV_1_ was positive in 53 (48.62%), PC_40_sGaw was positive in 85 (77.98%) patients, and an increased FeNO level was noticed in 67 (61.47%) individuals. In 51 (46.79%) patients, the methacholine test was deemed positive by spirometry and plethysmography. The difference in the tests regarding asthma was statistically significant (Table 3).

The sensitivity, specificity, positive predictive value (PPV), negative predictive value (NPV), odds ratio for a positive test, and odds ratio for a negative test were calculated for the BDT, PC_20_FEV_1_, PC_40_sGaw, and FeNO in the diagnosis of asthma. A PC_40_sGaw had the highest sensitivity to the diagnosis of asthma (97%). The BDT demonstrated the highest specificity (94%). The specificity for a PC_20_FEV_1_ was somewhat lower, at 0.88. The positive predictive values were high for all four tests, including 92% for a PC_20_FEV_1_, 90% for the BDT, 87% for a PC_40_sGaw, and 82% for FeNO ≥25 ppb. A PC_40_sGaw had the highest negative predictive value (92%). The highest odds ratio for a positive test was for a PC_20_FEV_1_; it was 5.32. The highest odds ratio for a negative test was for the BDT, with a value of 0.8. The results for the BDT, the BPT assessed by spirometry, and FeNO are presented in Table 4.

Since the optimal value for sGaw in the diagnosis of airway hyperreactivity is not clearly determined, we compared three potential values of sGaw: a PC_40_sGaw, PC_45_sGaw, and PC_50_sGaw. As expected, a PC_40_sGaw showed the highest sensitivity, while a PC_50_sGaw had the highest specificity. The best ratio of sensitivity, specificity, PPV, NPV, and odds ratio was for a PC_45_sGaw. The results for a PC_40_sGaw, PC_45_sGaw, and PC_50_sGaw are presented in Table 5.

When a PC_40_sGaw was combined with FeNO, the sensitivity was 71%, the specificity was 85%, the positive predictive value was 92%, the negative predictive value was 56%, the odds ratio for a positive test was 4.69, and the odds ratio for a negative test was 0.34 in the diagnosing of asthma.

## 4. Discussion

The aim of our study was to evaluate the diagnostic value of plethysmography compared to spirometry in the assessment of AHR to the methacholine challenge test (MCT) in patients suspected of having CVA. The results of our study showed that the PC_40_sGaw is more sensitive than the traditionally used PC_20_FEV_1_ (97% vs. 64%) in response to an MCT, but has a lower specificity (67% vs. 88%). The mean provocation concentration of methacholine that caused a PC_40_sGaw was significantly lower than for a PC_20_FEV_1_ (0.5 vs. 1.0 mg/mL). When a PC_45_sGaw was used as a diagnostic criterion, the specificity of the test increased (82%), while maintaining a high sensitivity (88%). These results suggest that plethysmography could be a valuable tool in the assessment of AHR in response to the MCT.

Cough variant asthma (CVA) is a special type of asthma characterized by the presence of chronic cough as the sole symptom. In accordance with the definition of CVA, the participants in our study had normal baseline values for spirometry and plethysmography. When lung function parameters were compared between the asthma and non-asthma groups, the asthma group had considerably lower spirometric and sGaw values. Although this difference reached statistical significance, it had no clinical significance and could not guide the diagnosis. CVA is considered to share the same pathogenesis and pathohistological process as conventional asthma, as follows: eosinophilic inflammation, bronchial hyperreactivity, and, if left untreated, symptoms can progress to conventional asthma and, eventually, to airway remodeling [5]. Thus, it is not unexpected that lung function is lower in CVA patients compared to healthy subjects. Asthma is a condition in which typically the degree of airway obstruction is variable. Asthma shows many different phenotypic features and lung function is only one of them, while others involve an inflammatory component with different cytological and molecular presentations. In recent years this spectrum of different phenotypes and endotypes has been recognized, so nowadays asthma is not considered as a single disease but as an asthma syndrome.

In our study, out of 76 patients in whom asthma was confirmed, only 19 had a positive BDT. The sensitivity of the BDT in our study was low (25%), but the specificity was very high (94%). Our results indicate that the sensitivity of the BDT is low in patients with asthma and normal spirometric values; however, if a 20% improvement in FEV_1_ is achieved despite normal values, the finding indicates asthma with high certainty.

Other studies including asthmatic individuals with normal initial spirometry also indicate the low sensitivity of the BDT. Goldstein MF et al. reported a BDT sensitivity of only 6.12% in a study of the population with suspected asthma with normal findings on lung examination, chest radiography, and baseline spirometry. The absence of reversibility on the BDT did not exclude asthma, as there was a high rate of false negative results (79.31%) [8]. Hunter CJ et al. reported the FEV_1_ response after the BDT in a mixed group of healthy subjects, patients with mild asthma, and non-asthmatics with asthma-like symptoms. The sensitivity of the post-BDT response was 49%, while the specificity was 70% [25].

When asthma is suspected and the BDT is negative, it is reasonable to perform a BPT. The test is generally considered to have high sensitivity and negative predictive value, while having a moderate specificity and relatively low positive predictive value [26]. This means that a negative BPT rules out the diagnosis of asthma with high certainty. A positive BPT does not automatically confirm the diagnosis of asthma because AHR can also be present in other entities such as allergic rhinitis, cystic fibrosis, and chronic obstructive pulmonary disease. In our study, the specificity of a PC_20_FEV_1_ was higher (88%) than the sensitivity (64%). Data on high sensitivity and moderate specificity are primarily the result of epidemiological studies. The results of some clinical studies, on a selected patient population, are in line with our data. In a study involving suspected asthmatics with normal baseline lung function, the sensitivity of the MCT was 85.71%, and there were no false-positive results (specificity 100%) [8]. Siersted HC et al. showed a sensitivity of 69% for airway responsiveness to methacholine in a population-based sample of healthy adolescents and subjects with asthma or at risk of developing asthma [27]. Hunter CJ et al. reported that in asthmatic adults who have normal or near-normal spirometric values, the MCT and differential sputum eosinophil counts are the most important tests for differentiating asthma from pseudo-asthma. Both the sensitivity and specificity of the MCT were high (≥90%) [25]. The pre-test probability of the MTC increases if symptoms consistent with asthma are present at the time of testing [12,28]. Higgins BG et al. showed that a positive MCT was more likely to be present in those with a history of wheezing and dyspnea, while it was weakly associated with a history of day or night cough [29]. Given that CVA symptoms are nonspecific and spirometric values are normal, the pre-test probability of the MCT in CVA diagnosis is lower than in patients with conventional asthma.

It was reported that in asthma patients with normal lung function, the sensitivity of an MCT determined by FEV_1_ alone increased by 37% when FVC, sGaw, and thoracic gas volume (TGV) evaluations were added to the analysis [30]. We explored the hypothesis that assessing nonspecific bronchial hyperreactivity using plethysmography would improve the diagnostic value of the MCT. When we used a PC_40_sGaw to define AHR, the sensitivity of the bronchoprovocation test increased significantly. The sensitivity of a PC_40_sGaw was 97%, while the specificity was 67%. According to our results, individuals with a positive PC_40_sGaw had a 2.91 times higher risk of having asthma than those who tested negative. The results of sGaw sensitivity in our study are entirely consistent with the results of Goldstein et al. [30]. Other authors have also demonstrated that plethysmography is more sensitive than spirometry for assessing the MCT. Parker et al. found that 22% of patients with methacholine-induced airway hyperresponsiveness had a decrease of PC_40_sGaw with no significant change in FEV_1_ [17].

Obviously, an ideal BPT would have a high level of sensitivity and specificity. Therefore, we investigated other cut-off values for sGaw to assess their diagnostic significance. As expected, increasing the cut-off value for sGaw increased specificity while decreasing sensitivity. As a result, we believe that the best ratio of specificity-to-sensitivity is at a PC_45_sGaw. The sensitivity and specificity of a PC45sGaw were 88% and 82%, respectively. Subjects with a positive test using a PC_45_sGaw had a 4.84 times greater risk of having asthma than subjects with a negative test. Among the subjects in our study in whom asthma was clinically suspected, AHR was proven in 83.5%. We explain such a high percentage of confirmed hyperreactivity by the fact that a detailed medical history was taken, a thorough diagnostic work-up was carried out to exclude other causes of cough (including an examination by an otorhinolaryngology specialist), and several tests for AHR detection were used.

Plethysmography’s greater sensitivity in detecting bronchial hyperreactivity in CVA compared to spirometry can be attributed to a variety of causes. Body plethysmography gives comprehensive information about the respiratory system. It provides information on lung residual volume (RV), total lung capacity (TLC), intrathoracic gas volume (ITGV), and specific airway resistance (sRaw). While FEV_1_ reflects the mechanical properties of the large and medium-sized airways, sRaw provides information on the entire airway. Another advantage of body plethysmography is that it allows the measurement of airway resistance while avoiding a forced respiratory maneuver [22]. Slats AM et al. showed that maximal lung inflation to total lung capacity (TLC), as achieved by taking deep inhalations, has a bronchodilating and bronchoprotective effect [31]. Lung inflation has a stretching effect on the airways, especially the noncartilaginous ones. Inspiration to TLC and forced expiratory maneuvers widen the airway diameter, reducing the respiratory airflow resistance [32]. Back in 1981, Fish JE et al. showed that deep inspiration has a greater bronchodilation effect in non-asthmatics than in asthmatics [33]. These observations have been confirmed by several studies, including a very recent one using a protocol similar to ours in subjects who underwent a methacholine BPT for various reasons including cough [16]. In healthy subjects, deep inspiration maneuvers performed within a few minutes before the methacholine administration protected the airways from bronchoconstriction. In patients with mild to moderate asthma, deep inspiration was able to partially reverse bronchial obstruction [34]. This protective mechanism is weakened in patients with severe asthma. Airway inflammation, remodeling, and peripheral bronchoconstriction prevent airway smooth muscles from stretching [35]. Thus, the mechanism for less FEV_1_ than sGaw response in our challenge protocol could be explained by the transient bronchodilator effect of deep inhalation preceding the forced expiratory maneuver. The bronchodilatory effect of deep inspiration in asthma is positively associated with inflammation in airway smooth muscle cells and submucosa on bronchial biopsies [31]. Since CVA represents a milder form of asthma with ill-defined symptoms, less inflammatory infiltration of the airways is expected. The use of calm breathing to assess bronchial hyperreactivity in CVA may be more important than in conventional asthma. When spirometry is used in the assessment of AHR, the basic prerequisites for a successful test largely depend on the patient’s ability to perform acceptable spirometric maneuvers, as well as the subject’s cooperation and compliance. It also depends on the respiratory technician’s ability to instruct and motivate the patient to properly perform spirometry. It is easier for the patient to perform plethysmography correctly because only tidal breathing is required. We have demonstrated that plethysmography enables the confirmation of AHR by using a lower concentration of methacholine compared to spirometric assessment. Therefore, the test is completed in fewer steps, making it less demanding for the patient and the respiratory technician. Given that inhaling methacholine can occasionally result in bronchospasm, the possibility of completing the test using a lower dosage of methacholine is safer for the patient.

Many clinicians include FeNO as an indirect measure of airway inflammation in their diagnostic algorithms. Numerous studies have reported that FeNO levels are elevated in asthma patients regardless of the severity of the disease [36,37]. In our study, the median FeNO levels were higher in the asthma group than in the non-asthma group, but were rather high in both groups. This may be explained by nasal contamination by exhaled NO due to the use of nose clips, and may be the reason for the low diagnostic value of FeNO. Recently, Chen LC et al. reported that that combining FeNO with other spirometric values can greatly improve CVA prediction compared to using either alone [38]. We also analyzed the sensitivity and specificity of FeNO in combination with a PC40sGaw; however, the combination did not improve the diagnostic value of FeNO.

There are several limitations of our study. The asthma group was older than the non-asthma group, which could be a confounding factor affecting AHR. Nevertheless, the influence of age on bronchial hyperresponsiveness is not entirely clear. A review that included eighteen studies, published between 1983 and 2002, showed a positive association between age and AHR [39]. However, this relationship does not appear to be linear. A cross-sectional study involving 148 healthy non-smokers aged 5 to 86 years showed that the bronchoconstriction response was more pronounced in the youngest and oldest age groups, in contrast to the middle age group [40]. The reasons for increased airway hyperreactivity in old age are multiple. Inflammatory and neuronal mechanisms, the influence of atopy, as well as the appearance of asthma in old age should be taken into account. Also, reduced lung function has been identified as one of the most important determinants of bronchial hyperreactivity, partly due to geometric factors, but also smoking history [39]. Smoking is a known risk factor for bronchial hyperreactivity [41,42]. In our study, patients were instructed not to smoke 24 h before testing in order to reduce the influence of smoking. Although smoking habits did not differ significantly between AHR and non-AHR, nor between asthma and non-asthma groups, 24 h abstinence from smoking may not completely eliminate smoking’s impact on test results. Therefore, in future research, smokers should be completely excluded. Another drawback of our study is that it was retrospective, which precluded detailed phenotyping. In future research, it would be useful to precisely determine the phenotype of the patient in order to evaluate whether some phenotypic characteristics, such as the type of inflammation, duration of cough, or age of symptom onset, could influence AHR detection.

Our study’s strength may be that we only included treatment-naïve patients. Study participants were evaluated by a pulmonologist in a specialized pulmonology outpatient clinic, and only those with suspected CVA were included in the study. Upper respiratory tract etiology, gastroesophageal reflux disease, and various intrathoracic causes of cough were excluded. Study participants underwent an extensive evaluation of AHR. In those with AHR, anti-asthma treatment was started. Diagnosis of asthma was confirmed based on the symptoms, course of treatment, and response to anti-asthma therapy after one year of follow-up.

## 5. Conclusions

CVA is a common cause of chronic cough. It is characterized by nonspecific symptoms and normal values of basic spirometry. Clinicians most often use the BDT and MCT performed by spirometry to prove AHR. In CVA, the BDT is often negative due to the absence of evident airway obstruction, and the MCT performed by spirometry is of insufficient sensitivity. Therefore, it is necessary to use sensitive and specific diagnostic methods that will facilitate the diagnosis of this type of asthma in order to avoid misdiagnosis and overdiagnosis. In our study, we demonstrated that an MCT performed by plethysmography has high sensitivity and specificity in the diagnosis of CVA. As we increased the cut-off values for the PCsGaw, the sensitivity of the test increased. For the diagnosis of AHR in individuals with suspected CVA, we believe that defining a positive MCT as a 45% drop in sGaw (PC45sGaw) provides the highest sensitivity and specificity ratio. In our opinion, in patients with suspected CVA, a negative BDT and a BPT assessed by spirometry, performing additional testing that would include a BPT with plethysmography would be useful.

## Figures and Tables

**Table 1:** The baseline data in the asthma and non-asthma group.

	Asthma (*n* = 76)		Non-Asthma (*n* = 33)			
Parameter	Value	SD/QD	Value	SD/QD	t/U	*p*-Value
anthropometric data						
Height, male (cm), mean	179.35	8.3	178.46	5.27	0.35	0.731
Height, female (cm), mean	167.28	6.93	168.25	7.74	0.51	0.608
Weight, male (kg), mean	84.96	16.76	83.00	13.34	0.36	0.731
Weight, female (kg), mean	68.38	10.27	64.20	10.21	1.55	0.125
BMI (kg/m^2^), mean	25.06	4.04	24.12	4.41	−1.09	0.278
lung function data						
FVC (L), mean	4.19	0.86	4.54	1.05	1.83	0.070
FVC %, median	104.5	106.15	108.0	107.25	1133.5 *	0.426
FEV_1_ (L) mean	3.35	0.71	3.85	0.90	3.11	0.002
FEV_1_ %, median	99.0	98.65	105.0	108	779 *	0.002
FEV_1_/FVC, mean	80.05	5.9	84.92	5.14	4.11	<0.001
MEF_50_ (L), median	3.52	3.51	4.32	4.28	787 *	0.002
MEF_50_ %, mean	77.6	19.2	91.0	20.1	3.6	0.001
RV (L), median	2.05	2.01	1.67	1.75	727 *	0.001
RV %, median	116.1	115.65	104.9	105.8	747 *	0.001
TLC (L), mean	6.21	1.16	6.11	0.99	−0.43	0.667
TLC %, median	107.0	105.9	99.8	103.2	936.5 *	0.036
RV/TLC %, median	105.8	108.5	102.2	102.7	914 *	0.025
sGaw (L/(kPa/s)), mean	0.97	0.2	1.13	0.27	3.55	0.001
sGaw %, mean	98.4	18.3	115.0	27.0	3.7	<0.001

Normally distributed continuous data are presented as mean (SD). Abnormally distributed data are presented as median (QD). * indicates Mann–Whitney U test. Abbreviations: BMI, body mass index; FVC, forced vital capacity; FEV_1_, forced expiratory volume in 1 s; MEF_50_, forced expiratory flow at 50% of forced vital capacity; RV, residual volume; TLC, total lung capacity; sGaw, specific airway conductance.

**Table 2:** Comparison of pre- and post-test lung function parameters in patients with and without asthma.

Test		Asthma (*n* = 76)		Non-Asthma (*n* = 33)		*p*-Value for Interaction
	Parameter	Pre	Post	Pre	Post	
BDT	FEV_1_ (L)	3.35 ± 0.71	3.59 ± 0.75	3.85 ± 0.9	4.00 ± 0.92	0.009
	FEV_1_ (%)	100 ± 11	107 ± 5	107 ± 11	104 ± 4	<0.001
	FVC (L)	4.2 ± 0.9	4.3 ± 0.9	4.5 ± 1.1	4.6 ± 1.0	0.07
	FVC (%)	106 ± 9	104 ± 5	105 ± 8	102 ± 4	0.23
BPT (PC_20_)	FEV_1_ (L)	3.4 ± 0.7	2.6 ± 0.6	3.9 ± 0.9	3.5 ± 1.0	<0.001
	FEV_1_ (%)	101 ± 12	78 ± 7	108 ± 12	88 ± 9	0.32
BPT (PC_40_)	sGaw (L/(kPa/s)	0.97 ± 0.20	0.46 ± 0.13	1.13 ± 0.27	0.81 ± 0.30	<0.001
	sGaw %	98 ± 18	48 ± 8	115 ± 27	71 ± 17	0.18

Abbreviations: BDT, bronchodilator test; BPT, bronchial provocation test; FVC, forced vital capacity; FEV_1_, forced expiratory volume in 1 s; sGaw, specific airway conductance; PC_20_, provocative concentration causing a 20% fall in FEV_1_; PC_40_, provocative concentration causing a 40% fall in sGaw.

**Table 3:** The difference in the tests regarding asthma.

Test		Asthma				Total		X^2^	*p*
		No (*n* = 33)		Yes (n = 76)		*n* = 109			
		n	%	n	%	n	%		
BDT	negative	31	93.94	57	75.00	88	80.73	4.16	0.041
	positive	2	6.06	19	25.00	21	19.27		
PC_20_FEV_1_	negative	29	87.88	27	35.53	56	51.36	25.24	<0.001
	positive	4	12.12	49	64.47	53	48.62		
PC_40_sGaw	negative	22	66.67	2	2.63	24	22.02	51.29	<0.001
	positive	11	33.33	74	97.37	85	77.98		
FeNO	negative	21	63.64	21	27.63	42	38.53	11.12	0.001
	positive	12	36.36	55	72.37	67	61.47		

Abbreviations: BDT, bronchodilator test; PC_20_FEV_1_, provocative concentration causing a 20% fall in FEV_1_; PC_40_sGaw, provocative concentration causing a 40% fall in sGaw; FeNO, fractional exhaled nitric oxide; n, number; %, percentage; X^2^, chi-squared test.

**Table 4:** Diagnostic value of BDT, PC_20_FEV_1_, and FeNO.

	BDT	PC_20_FEV_1_	FeNO
Sensitivity (%)	25	64	72
Specificity (%)	94	88	64
PPV (%)	90	92	82
NPV (%)	35	52	50
Odds ratio for a positive test	4.13	5.32	1.99
Odds ratio for a negative test	0.80	0.4	0.43

Abbreviations: BDT, bronchodilator test; PC_20_FEV_1_, provocative concentration causing a 20% fall in FEV_1_; FeNO, fractional exhaled nitric oxide; PPV, positive predictive value; NPV, negative predictive value.

**Table 5:** Diagnostic value of PC_40_sGaw, PC_45_sGaw, and PC_50_sGaw.

	PC_40_sGaw	PC_45_sGaw	PC_50_sGaw
Sensitivity (%)	97	88	63
Specificity (%)	67	82	91
PPV (%)	87	92	94
NPV (%)	92	75	52
Odds ratio for a positive test	2.91	4.84	6.93
Odds ratio for a negative test	0.05	0.15	0.14

Abbreviations: PC_40_sGaw, provocative concentration causing a 40% fall in sGaw; PC_45_sGaw, provocative concentration causing a 45% fall in sGaw; PC_50_sGaw, provocative concentration causing a 50% fall in sGaw; PPV, positive predictive value; NPV, negative predictive value.

## Data Availability

The data presented in this study are available on request from the corresponding author.
